# CO_2_ Capture-Mineralization for Calcium-Looping Integrated with Methane Dry Reforming

**DOI:** 10.1021/acs.langmuir.5c02551

**Published:** 2025-09-08

**Authors:** Zhi Xuan Law, Nattanan Watcharasawat, Varong Pavarajarn, De-Hao Tsai

**Affiliations:** † Department of Chemical Engineering, 34881 National Tsing Hua University, Hsinchu 300044, Taiwan, R.O.C; ‡ Department of Chemical Engineering, 26683 Chulalongkorn University, Bangkok 10330, Thailand

## Abstract

Chemical absorption of carbon dioxide using monoethanolamine (MEA) is a well-established method for postcombustion CO_2_ capture. In this study, we aimed to integrate (1) the MEA-based CO_2_ capture with the regeneration of MEA using calcium-based mineralization, followed by (2) direct utilization of captured CO_2_ to form syngas via a calcium looping-based dry reforming of methane (CaL-DRM), an interfacial catalytic process. The results show that room-temperature CO_2_ capture-MEA regeneration was achievable by using calcium-based mineralization. The formed Ni–Ca material was shown to be active for converting the captured CO_2_ into syngas via the CaL-DRM reaction at 600 °C. A 10-cycle stability test confirmed the operational stability of the Ni–Ca material, with consistent CO_2_ uptake capacity (*X*_CO2_ = 6.1–6.3 mmol/g_sample_) and stable syngas yields (*Y*_H2_ = 14.2–14.5 mmol/g_sample_, *Y*_CO_ = 12.1–12.9 mmol/g_sample_). These results demonstrate the feasibility of integrating CO_2_ capture-mineralization with CaL-DRM, offering a sustainable and energy-efficient pathway for CO_2_ utilization and syngas generation.

## Graphical abstract

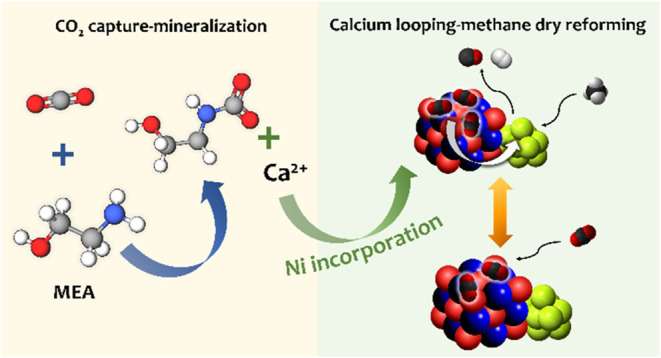

## Introduction

The increasing concentration of carbon dioxide in the atmosphere has emerged as one of the most critical environmental challenges in recent years.^1–4^ Anthropogenic activities, especially industrial processes have significantly contributed to the rise in CO_2_ levels, leading to global warming and climate change. Several carbon capture, utilization and storage (CCUS) strategies are being explored to reduce CO_2_ emissions.^5–7^ Among these, postcombustion CO_2_ capture has attracted significant attention due to its compatibility with existing industrial processes, allowing for relatively straightforward implementation.^5,8^

Postcombustion CO_2_ capture approaches typically involve the removal of CO_2_ through chemical or physical absorption processes.^3,5,7,9,10^ In chemical absorption, monoethanolamine (MEA) is one of the most widely studied solvents, owing to its high reactivity and selectivity toward CO_2_.^1,2,5,7,8,11–13^ MEA reacts with CO_2_ to form a carbamate intermediate, facilitating efficient separation from gas streams.^11^ The absorption of CO_2_ by MEA generally proceeds via the zwitterion mechanism. In this pathway, CO_2_ first reacts with the amine group to form a zwitterionic intermediate (eq 1).^14^ This intermediate is then stabilized by proton transfer with another base, typically a second MEA molecule, to form a stable carbamate (eq 2).^14^

1 $${\mathrm{CO}}_{2}+{\mathrm{RNH}}_{2}↔{{\mathrm{RNH}}_{2}}^{+}\text{−}{\mathrm{COO}}^{-}$$

2 $${{\mathrm{RNH}}_{2}}^{+}\text{−}{\mathrm{COO}}^{-}+{\mathrm{RNH}}_{2}↔{\mathrm{RNHCOO}}^{-}+{{\mathrm{RNH}}_{3}}^{+}$$

However, the practicality of MEA absorption is limited by challenges such as solvent degradation and the high energy requirements for thermal regeneration.^1,2,11,15^

Consequently, developing effective recovery methods for CO_2_-absorbed MEA has become an important focus to enhance the economic feasibility of the CO_2_ capture process.^6,8,16^ In this instance, recovering MEA through mineralization using a calcium-based source offers a promising alternative, producing stable carbonate products (i.e., CaCO_3_) that are not easy to decompose, which addresses storage safety concerns and enabling in situ downstream utilization of the captured CO_2_.^8,17–20^ In this process, the carbamate species formed undergo hydrolysis, regenerating the MEA and forming bicarbonate (eq 3). The resulting bicarbonate then reacts with calcium ions to precipitate stable calcium carbonate (eq 4).^20^

3 $${\mathrm{RNHCOO}}^{-}+H_{2}O↔{\mathrm{RNH}}_{2}+{{\mathrm{HCO}}_{3}}^{-}$$

4 $${\mathrm{Ca}}^{2+}+{{\mathrm{HCO}}_{3}}^{-}\to {\mathrm{CaCO}}_{3}+H^{+}$$

The CaCO_3_ formed after MEA recovery can either be stored or utilized in downstream applications. In a reversible calcium looping (CaL) process, CaCO_3_ is calcined to release CO_2_, regenerating it back into CaO, which can be reused to adsorb CO_2_.^21–25^ However, conventional CaL approaches typically require calcination temperatures >900 °C for effective CaCO_3_ regeneration.^23,24,26,27^ This high-temperature process often leads to significant sintering of CaO, resulting in a gradual decline in its CO_2_ uptake capacity over multiple cycles.^21,24,28^ In contrast, regeneration via integrated carbon capture and utilization (ICCU) offers a promising alternative by substantially lowering the required regeneration temperature.^29^ This approach enables simultaneous CaCO_3_ decarbonation and direct CO_2_ utilization, minimizing energy consumption and mitigating material sintering.^24,28,30^

Among ICCU applications, the integration of calcium looping with methane dry reforming (DRM) has been extensively studied due to its potential for efficient CO_2_ utilization and syngas production.^22,23,28,31–36^ In a typical CaL-DRM process, Ni–CaO-based materials are employed as dual-functional materials, where CaO acts as the CO_2_ sorbent in the first step to capture CO_2_ through carbonation, and Ni serves as the active metal in the second step to catalyze the DRM reaction.^37–40^ In this case, by incorporating Ni into the CaCO_3_ material obtained from MEA absorption, the calcium source can potentially be regenerated, and the CO_2_ can be effectively utilized through the CaL-DRM approach.

The main objective of this study is to develop a tandem process that integrates CO_2_ capture-mineralization with the CaL-DRM process. As illustrated in Scheme 1, CO_2_ is first captured using MEA, forming a MEA-CO_2_ solution. This solution is then reacted with a calcium source to form CaCO_3_, allowing MEA to be recovered and reused. The obtained CaCO_3_ is subsequently incorporated with Ni and employed as a dual-functional material (Ni–Ca) in the CaL-DRM process. During this process, methane is introduced, and the Ni–Ca material facilitates both syngas production and CaO regeneration, enabling continuous reuse in subsequent cycles. Overall, the mineralization approach appears to be an effective way to prepare the CaO-based material, and since CaCO_3_ is typically regarded as a waste byproduct from the absorption–mineralization process, utilizing it in the CaL-DRM process can be considered as a form of recycling, allowing the captured carbon to be reused directly in the downstream CO_2_ conversion. This reduces the need for additional CaO preparation, which is energy-intensive, and also minimizes raw material consumption, thereby improving both the energy efficiency and overall sustainability of the process. To the best of our knowledge, this study is the first to explore the integration of CO_2_ capture-mineralization with CaL-DRM, thus offering a novel and sustainable approach for enhancing CO_2_ utilization and facilitating the continuous recycling of the calcium sorbent.

**Scheme 1:**
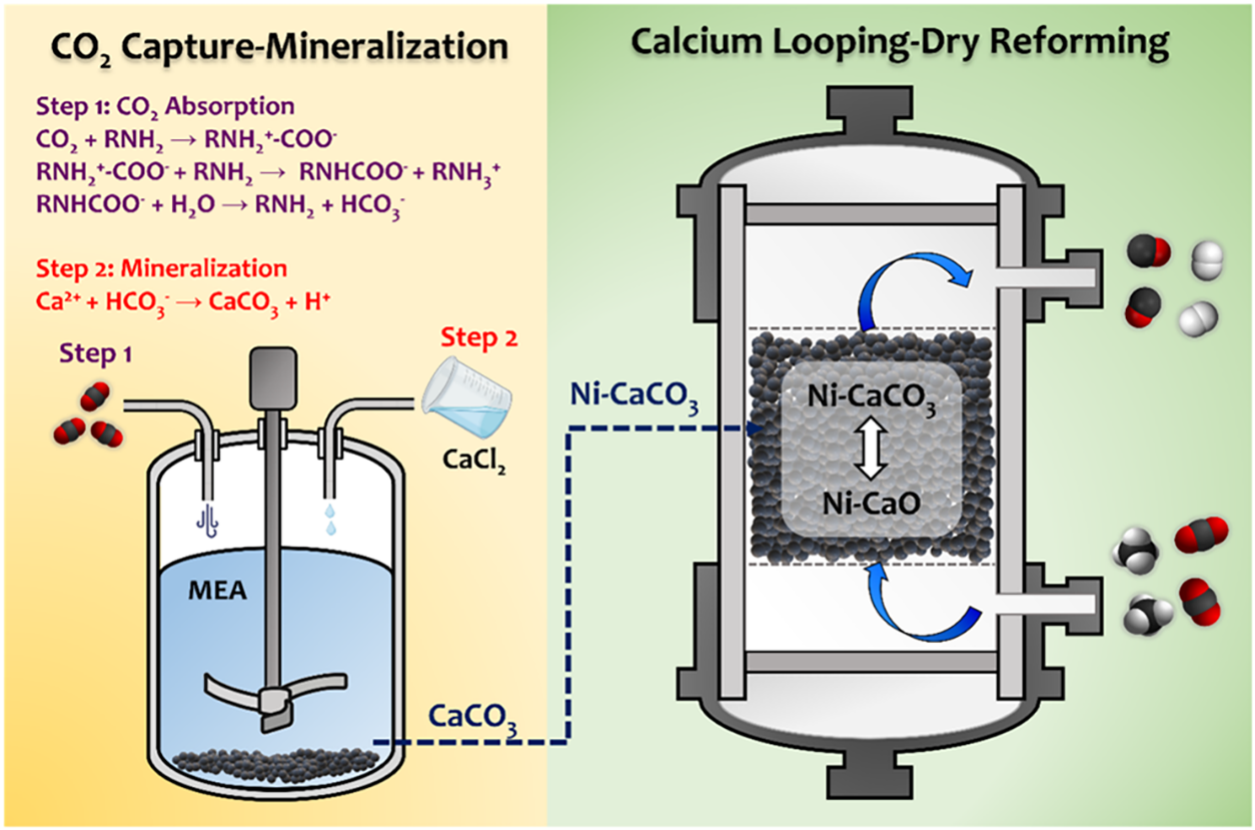
Integration of CO_2_ Capture-Mineralization Process (Left) with the Calcium Looping-Based Methane Dry Reforming (Right)

## Experimental Section

### Materials

Monoethanolamine (MEA) solution was prepared using ethanolamine (>98%; Thermo Fisher Scientific, Massachusetts). Calcium chloride (granular; Showa Chemical Industry, Tokyo, Japan) was utilized as calcium source for the mineralization process. Nickel­(II) nitrate hexahydrate (>98%; Showa Chemical Industry) was used as the nickel precursor. Deionized (DI) water (18.2 MΩ·cm; Millipore, Massachusetts) was used to prepare the MEA and CaCl_2_ solutions.

### Materials Characterization

The morphologies of both the as-prepared and spent materials were examined using a field emission scanning electron microscope (FE-SEM; Hitachi SU8010; Hitachi, Tokyo, Japan), and the elemental distribution was analyzed by energy-dispersive spectroscopy (EDS). A high-resolution transmission electron microscope (HR-TEM; JEM-2100HT; JEOL, Tokyo, Japan) with EDS operating at 300 kV was also employed to provide the microscopic images with higher resolution. X-ray diffraction (XRD) measurements were carried out with a powder X-ray diffractometer (D8 Advance, Bruker, Karlsruhe, Germany) using Cu–Kα radiation (λ = 1.5406 Å) at 40 kV and 25 mA. Inductively coupled plasma optical emission spectrometry (ICP-OES) was conducted to examine the fractional amounts of Ni in the sample. Five mL of HCl was used for the dissolution of sample overnight, and DI water was used for the subsequent dilutions.

CO pulse chemisorption was used to evaluate the metal dispersion (*D*_metal_) and metal surface area (*S*_msa_) of the samples using a temperature-programmed chemisorption system (ASAP 2920; Micrometrics, Norcross, GA). Approximately 0.1 g of the sample was first preheated in helium at 200 °C for 30 min. After cooling to 35 °C, 0.5 mL CO pulses were introduced repeatedly until no further CO uptake was detected. *D*_metal_ was calculated based on the total amount of CO adsorbed per gram of sample. In addition, CO_2_ temperature-programmed desorption (CO_2_-TPD) was carried out to assess the surface basicity using the same system. After pretreatment, CO_2_ pulses were injected at 35 °C until saturation, followed by desorption up to 800 °C at a heating rate of 10 °C/min.

### CO_2_ Capture and Mineralization

CO_2_ capture process was conducted by introducing CO_2_ at a flow rate of 250 mL/min into a 5 M MEA solution using a sparger. The solution was continuously stirred throughout the process to ensure effective gas–liquid contact. To minimize solvent loss, a filter was placed at the outlet to prevent evaporation. CO_2_ was bubbled through the MEA solution for approximately 2 h, and the solution’s pH was monitored. The absorption process was carried out until the pH stabilized (∼8), corresponding to a CO_2_ loading of approximately 0.78 ± 0.08 mmol of CO_2_ per mmol of MEA, as quantified by cumulative gas uptake measurements (i.e., detailed results shown in Figure 1). The CO_2_ consumption rate and cumulative CO_2_ uptake results were obtained from three replicate measurements, with error bars representing one standard deviation.

**Figure 1:**
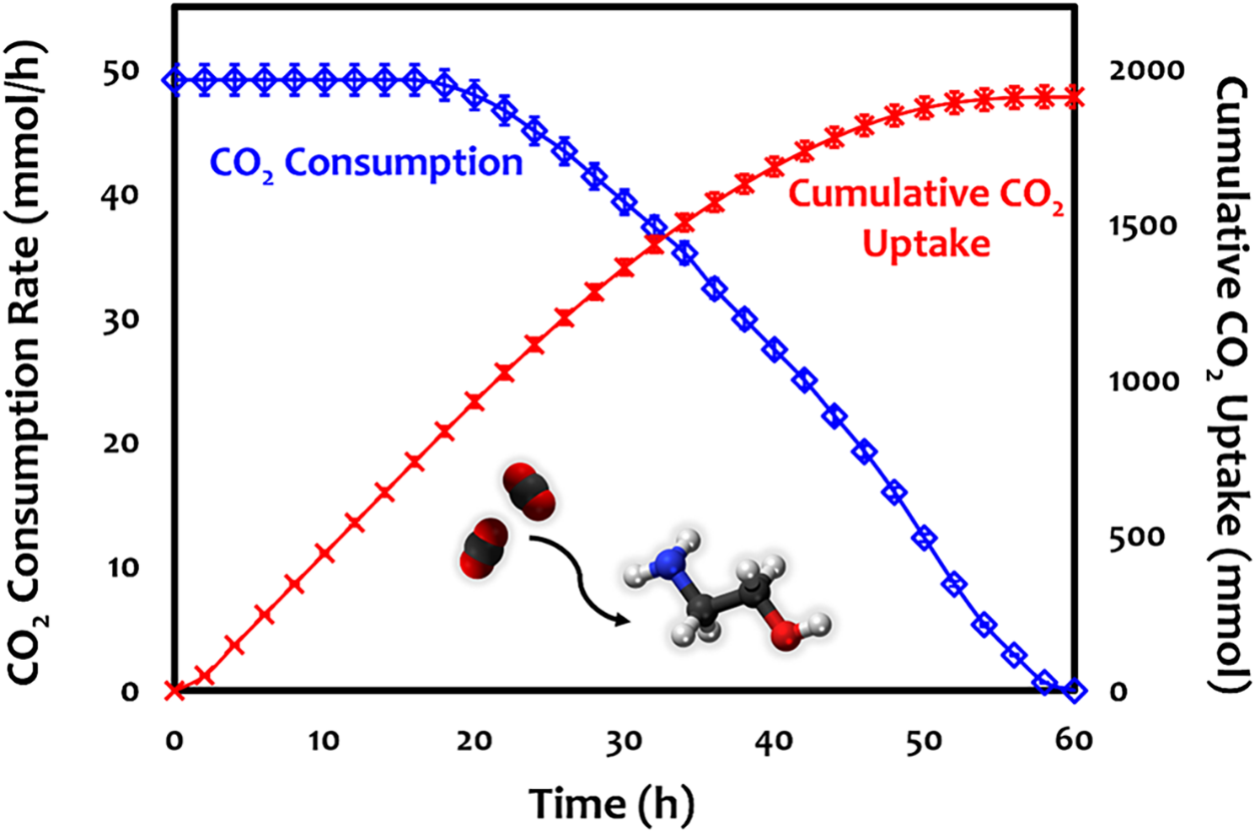
CO_2_ consumption rate and cumulative CO_2_ uptake over time during absorption using MEA.

In the mineralization process, the CO_2_-absorbed MEA solution and a 5 M CaCl_2_ solution were mixed at a 1:2 volume ratio and stirred continuously for 3 h to ensure uniform mixing. After that, the mixture was centrifuged to separate the solid phase from the liquid. The liquid phase was discarded, and the remaining solid was collected and dried in an oven at 60 °C overnight to obtain the final product.

### Calcium Looping-Based Methane Dry Reforming

The catalytic performance of the calcium looping-based dry reforming of methane (CaL-DRM) was evaluated using a self-established fixed-bed reactor system (i.e., as illustrated in Figure S1 of Supporting Information). For the preparation of catalyst, nickel nitrate was added directly via incipient wetness impregnation to the CaCO_3_ particles generated from the mineralization process, achieving a CaCO_3_/Ni weight ratio of 1:0.2. The amount of sample loaded for each performance test was 150 mg. For a temperature-programmed scanning test, total feed flow rate of 200 mL/min consisting 5 vol % CO_2_/N_2_ and 5 vol % CH_4_/N_2_ were used for the carbonation and DRM steps, respectively. The operating temperatures ranged from 30 to 600 °C for carbonation step and 550 to 800 °C for the DRM step. Cyclic stability was evaluated isothermally at 650 °C. Except for the first cycle, which began with a DRM step, each subsequent cycle consisted of a 15 min carbonation step in 5 vol % CO_2_/N_2_, followed by a 15 min DRM step in 5 vol % CH_4_/N_2_, with a 2 min N_2_ purge in between. The outlet concentrations of reactants and products were quantified using a series of analyzers, including a CO_2_ nondestructive infrared spectrometer (ND-IR; CI-IR 20; Chang-Ai, Shanghai, China), a CH_4_ ND-IR (CI-IR 10; Chang-Ai), a CO ND-IR (CI-IR 10; Chang-Ai), and a H_2_ thermal conductivity detector (TCD) analyzer (CI-TC 90; Chang-Ai).

Catalytic performances of the Ni–Ca material were expressed in terms of CO_2_ uptake capacity (*X*_CO2_) in the carbonation step, and H_2_ (*Y*_H2_) and CO yields (*Y*_CO_) in the DRM step using eqs 5, 6, and 7, respectively

5 $$X_{\mathrm{CO}2}\left(\mathrm{mmol}/g_{\mathrm{sample}}\right)=M_{\mathrm{CO}2}/W$$

6 $$Y_{H2}\left(\mathrm{mmol}/g_{\mathrm{sample}}\right)=M_{H2}/W$$

7 $$Y_{\mathrm{CO}}\left(\mathrm{mmol}/g_{\mathrm{sample}}\right)=M_{\mathrm{CO}}/W$$

Here, *M*_CO2_ (unit: mmol) is the quantity of CO_2_ consumed in the carbonation reaction, and *W* (unit: g) is the amount of catalyst. *M*_H2_ and *M*_CO_ (unit: mmol) indicate the amount of H_2_ and CO generated during the subsequent DRM step.

## Results and Discussion

### CO_2_ Capture and Mineralization

For the quantitative analysis of the CO_2_ capture rate using MEA over time, a total gas flow of 200 mL/min with 10 vol % CO_2_/N_2_ was continuously fed into a 500 mL solution of 5 M MEA. Figure 1 shows CO_2_ consumption rate and cumulative CO_2_ uptake over time during MEA-mediated CO_2_ absorption. During the first 20 h, CO_2_ was completely absorbed. Subsequently, the CO_2_ consumption rate gradually decreased and reached a saturation point at around the 60th hour. The total CO_2_ uptake value was approximately 0.78 ± 0.08 mmol_CO2_/mmol_MEA_, corresponding to an efficiency of 78%. The decline in absorption capacity can be attributed to the system approaching chemical equilibrium, which limits the complete conversion of MEA to its CO_2_-bound form. A detailed discussion on the mass transfer characteristics, including estimated Reynolds, Schmidt and Sherwood numbers, is provided in Section S1 of the Supporting Information. After mineralization using CaCl_2_, a mixture of regenerated MEA and CaCO_3_ was formed. The CaCO_3_ was separated by centrifugation and dried overnight to obtain the final product.

### Calcium Looping-Based Methane Dry Reforming

Temperature-programmed scanning tests were conducted to evaluate the catalytic performance of the mineralized material. In the first DRM cycle, Ni was incorporated into CaCO_3_ via impregnation of a precursor solution (CaCO_3_/Ni weight ratio of 1:0.2). The actual Ni content in the sample was confirmed by inductively coupled plasma-optical emission spectrometry (ICP-OES), showing a Ni weight percentage of approximately 15.8%, which is close to the designed value. To prevent premature decarbonation of CaCO_3_ (i.e., CO_2_ released from the adsorbent at ∼600 °C), no ex situ calcination was applied. During the DRM process, as the temperature increased, the Ni precursor (Ni­(NO_3_)_2_) decomposed into NiO, which was then reduced in situ to metallic Ni by the H_2_ produced during the reaction, and possibly also via the partial oxidation of CH_4_ ^41^ (i.e., additional CH_4_-based temperature-programmed reaction profiles are provided in Figure S5 of Supporting Information). Here, the results of CH_4_-based temperature-programmed reaction showed no CO generation, indicating that partial oxidation of CH_4_ was not dominant during the reduction of NiO to Ni. As shown in Figure 2a, CO_2_ started to be released from the material at ∼600 °C, while methane dry reforming initiated at ∼635 °C, evidenced by the decrease in CH_4_ concentration and the simultaneous production of syngas (H_2_ and CO). The higher H_2_ concentration compared to CO was attributed to the occurrence of methane cracking (CH_4_ → C + 2H_2_).^42,43^ Note that some CO_2_ was released directly from the material without being converted, probably due to a weak interface between Ni and CaCO_3_, as Ni was initially introduced in its precursor form. Overall, the gaseous concentration profiles confirm the feasibility of employing the mineralized material in a CaL-DRM process.

**Figure 2:**
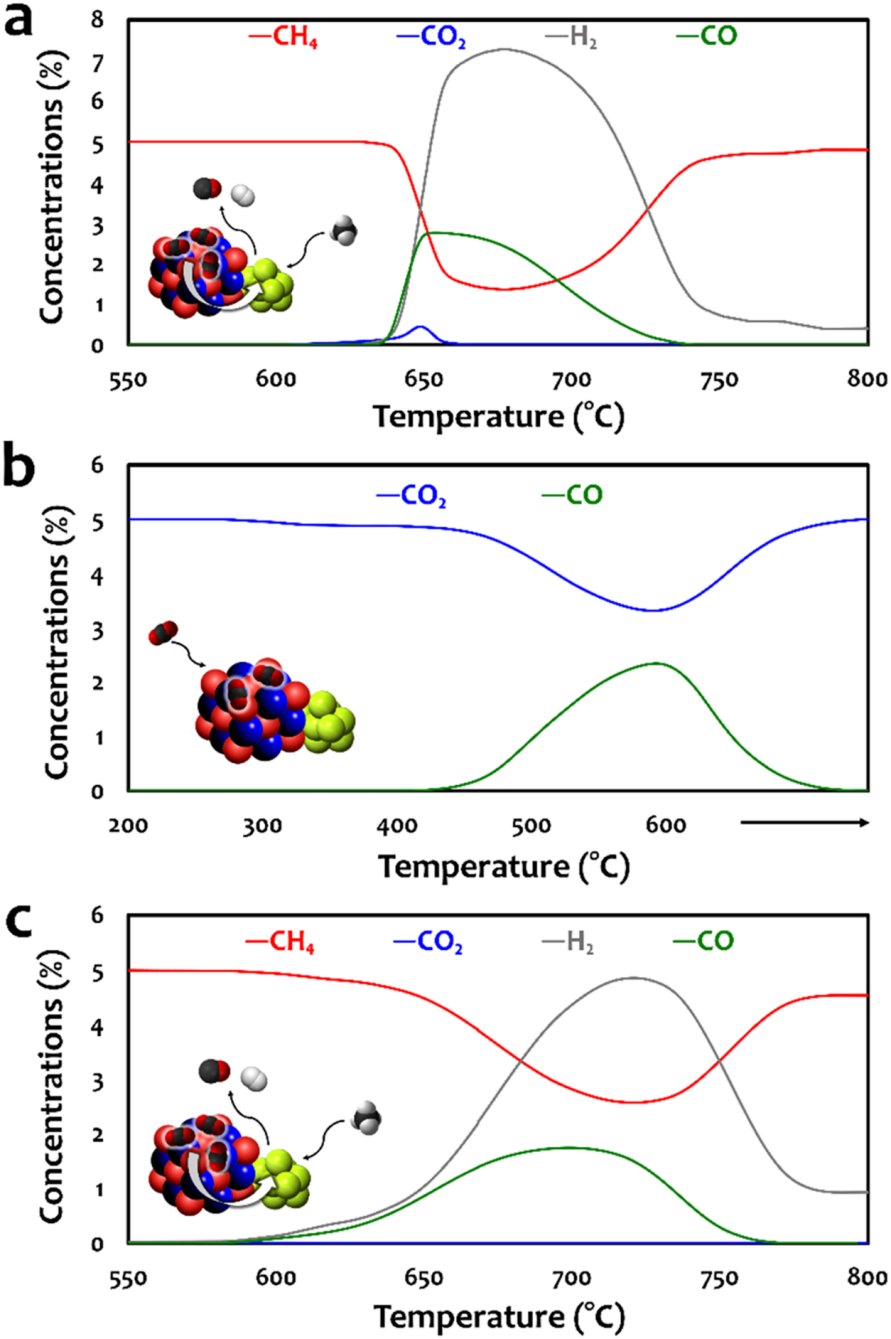
Gaseous concentration profiles during temperature-programmed catalytic performance test. (a) DRM using Ni-incorporated CaCO_3_ obtained from mineralization. (b) CO_2_ capture after DRM. (c) DRM after CO_2_ capture.

After the DRM process, the Ni–CaO was recarbonated to Ni–CaCO_3_ through direct CO_2_ capture. As shown in Figure 2b, the CO_2_ absorption profile shows a small peak at ∼330 °C, and achieved its highest consumption rate at ∼590 °C. Note that CO started to be released at ∼400 °C, with its concentration profile showing a similar trend as the CO_2_ consumption. This can be attributed to the occurrence of reverse Boudouard reaction (CO_2_ + C → 2CO),^42,43^ due to the significant coke deposition of the material arose from methane cracking during the previous DRM step. Overall, the CO_2_ capture capacity (i.e., excluding CO_2_ conversion via the reverse Boudouard reaction) was ∼6.3 mmol_CO2_/g_sample_. To better understand the CO_2_ capture behavior, the surface basicity of the sample after DRM was evaluated using CO_2_-TPD analysis, as shown in Figure S4. Two distinct desorption peaks at ∼365 and 540 °C were observed, corresponding to chemisorbed CO_2_ on basic sites associated with Ca­(OH)_2_ and CaO, respectively.^23,24,34^ The total surface basicity was determined to be approximately 2.63 mmol/g_sample_. These basic sites are essential in facilitating CO_2_ adsorption and contribute directly to the efficiency of the carbonation step in the CaL-DRM process.

The recarbonated Ni–CaCO_3_ was subjected to another DRM cycle, with the corresponding concentration profile shown in Figure 2c. Here, DRM initiated at ∼590 °C, and no CO_2_ was released from the adsorbent. The improved CO_2_ conversion efficiency can be attributed to the enhanced Ni–Ca interface, as the active Ni was already activated during the previous cycle. However, methane cracking remained significant, resulting in a H_2_/CO ratio of approximately 2. Figure S6 in the Supporting Information presents the H_2_/CO ratio profile, further supporting that methane cracking dominated at lower temperatures and became more pronounced as CO_2_ was gradually depleted.

Figure 3a shows the SEM image of mineralization-derived CaCO_3_, exhibiting an agglomeration of cubic structures, which is the characteristic of calcite phase. After the first DRM step (Figure 3b), the morphology became irregular due to the transformation of CaCO_3_ into CaO and the incorporation of Ni. EDS analysis confirmed the uniform distribution of Ni throughout the CaO surface, while the presence of carbon was attributed to methane cracking. After carbonation (Figure 3c), the deposited carbon was removed via the reverse Boudouard reaction, and CaO was reconverted into CaCO_3_ through CO_2_ capture. After the second DRM step, the structural changes became more pronounced, with the formation of carbon nanotubes resulting from methane cracking. Additional SEM images are provided in Figure S2 of the Supporting Information.

**Figure 3:**
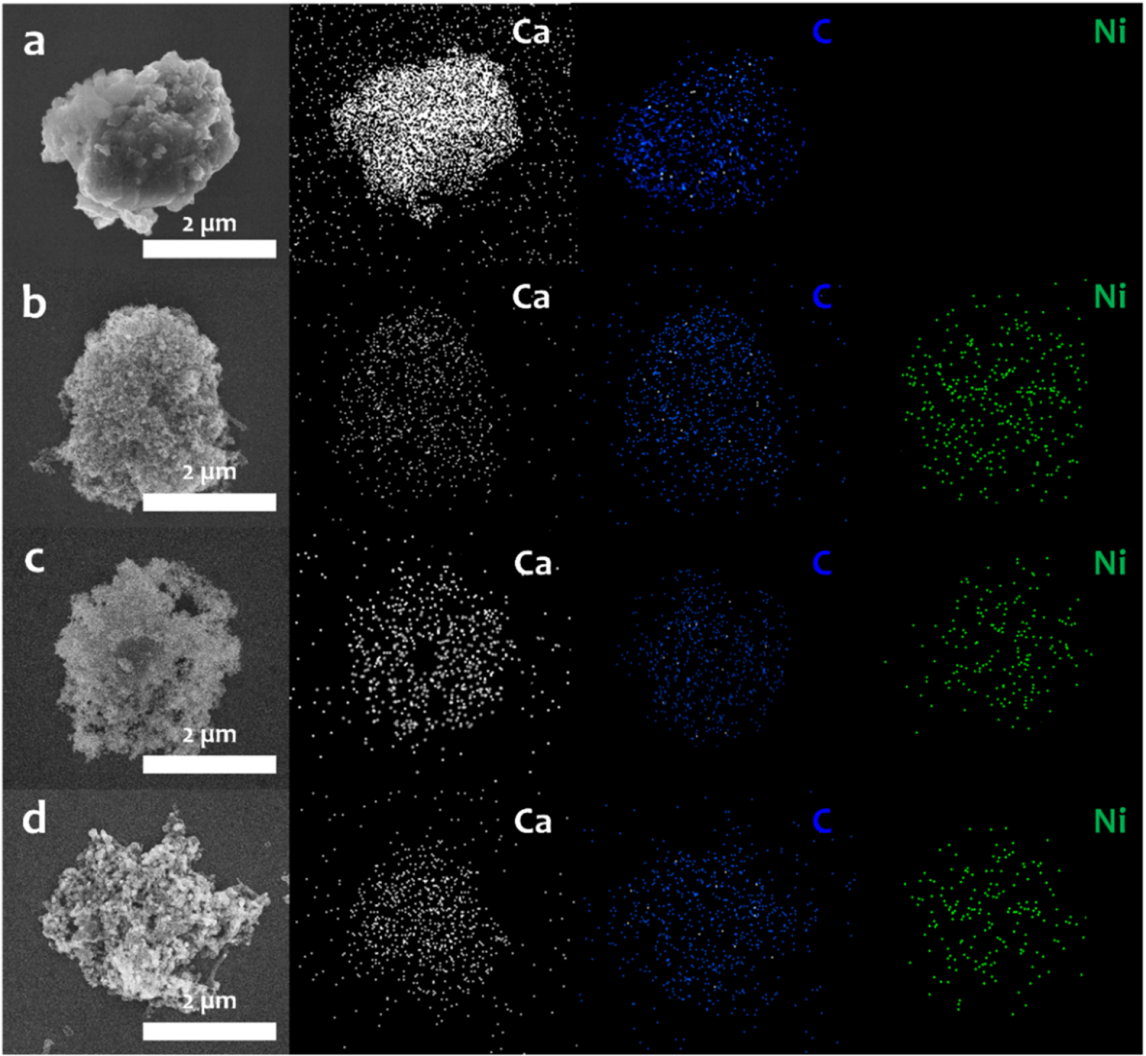
SEM-EDS images of the Ni–Ca material before and after temperature-programmed activity test. (a) CaCO_3_ after mineralization. (b) Ni–Ca material after first DRM step. (c) Ni–Ca material after carbonation step. (d) Ni–Ca material after second DRM step. The scale bars are 2 μm.

As shown in the HR-TEM images of the representative samples, the mineralization-derived CaCO_3_ (Figure 4a) exhibited a block-like morphology with clear lattice fringes characteristic of the calcite phase. After the first DRM step (Figure 4b), well-defined Ni–Ca interfaces were observed, indicating the successful in situ formation and dispersion of metallic Ni on the CaO surface during the reaction. In addition, distinct crystalline carbon structures with lattice fringes around ∼0.35 nm were also present, providing further evidence of methane cracking. The corresponding TEM-EDS images are provided in Figure S3 of the Supporting Information, confirming the uniform distribution of Ni across the CaO matrix.

**Figure 4:**
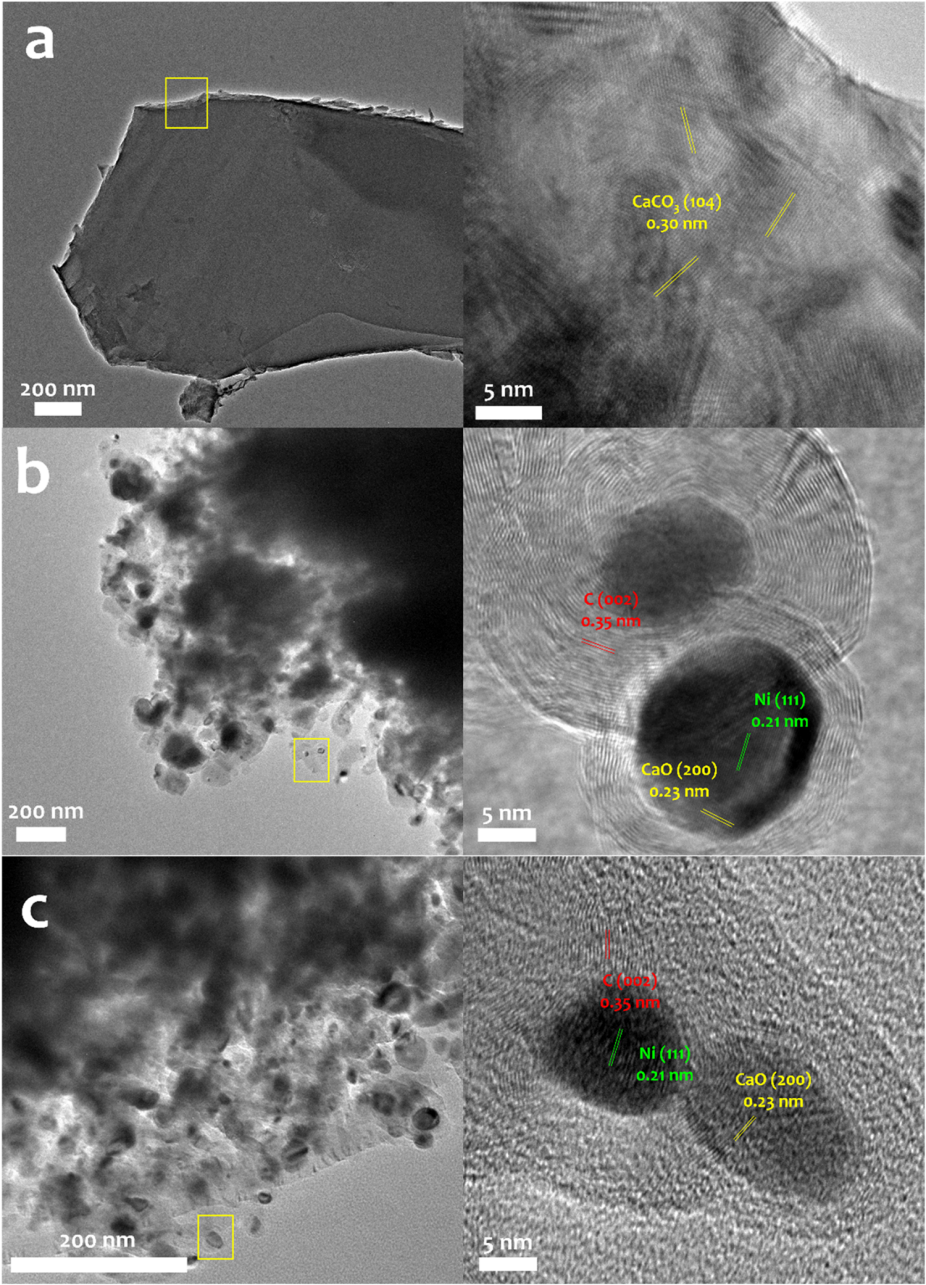
HR-TEM images of the representative Ni–Ca material before and after activity test. (a) CaCO_3_ after mineralization. (b) Ni–Ca material after first DRM step. (c) Ni–Ca material after 10-cycle stability test. The scale bars are 200 and 50 nm.

As shown in Figure 5, XRD analysis confirmed the presence of characteristic CaCO_3_ peaks in the calcite phase after mineralization (*d*_c,CaCO3_ = 35.2 nm). After DRM, the disappearance of these peaks indicated that most CaCO_3_ had decomposed into CaO. Meanwhile, Ca­(OH)_2_ was detected (*d*_c,Ca(OH)2_ = 8.8 nm), likely due to the hydration of CaO upon exposure to moisture. Ni characteristic peaks were observed (*d*_c,Ni_ = 21.7 nm), confirming the successful conversion of the Ni precursor into metallic Ni during the reaction. Additionally, a carbon crystallite phase was detected at 2θ = 26°, indicating coke deposition from methane cracking. After CO_2_ capture through CaO carbonation, most of the Ca­(OH)_2_ and CaO converted back to CaCO_3_, while Ni crystallites experienced sintering (*d*_c,Ni_ = 26.2 nm) due to the exposure to high temperature (i.e., 600 °C). After the second DRM step, CaCO_3_ completely decomposed into CaO and Ca­(OH)_2_, with further Ni sintering, resulting in an increased crystallite size of *d*_c,Ni_ = 29.3 nm.

**Figure 5:**
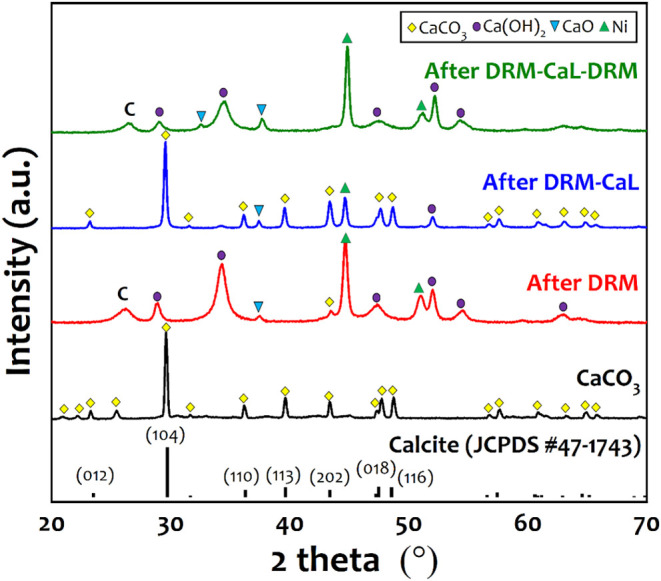
XRD pattern of the Ni–Ca material before and after temperature-programmed activity test.

### Mineralization of Ni–Ca Material after DRM

To further evaluate the feasibility of reconverting CaO to CaCO_3_ via mineralization, the Ni–Ca material obtained after the first DRM step (i.e., as shown in Figure 2a) was subjected to MEA-CO_2_ treatment. After mineralization, Ni–CaO was successfully transformed into Ni–CaCO_3_, as evidenced by the XRD pattern in Figure S8 of the Supporting Information, where all the CaO and Ca­(OH)_2_ characteristic peaks disappeared and converted to CaCO_3_. Besides, no Ni sintering was observed (*d*_c,Ni_ = 21.7 nm), owing to the absence of thermal exposure during the mineralization process. SEM analysis (Figure S9a) revealed that the remineralized Ni–Ca material exhibited a smoother surface compared to the recarbonated sample (i.e., as shown in Figure 3c), attributable to the lack of high-temperature treatment. However, the final yield of the remineralized product was reduced by approximately 30%, due to the additional separation and drying steps involved in the process.

The remineralized material was then subjected to another DRM step, with the concentration profile shown in Figure S7. Similar to the recarbonated sample (i.e., as shown in Figure 2c), DRM initiated at ∼590 °C, and no CO_2_ was released from the material. However, the amount of CH_4_ converted, as well as the H_2_ and CO generated, was significantly lower compared to the recarbonated sample, due to the incomplete conversion of captured CO_2_. As shown in the XRD pattern (Figure S8), after the DRM step, residual CaCO_3_ was detected, suggesting incomplete decomposition to CaO, likely due to the severe structural changes induced by the mineralization process. Meanwhile, Ni crystallites exhibited slight sintering, increasing to *d*_c,Ni_ = 23.2 nm. Overall, these results suggest that calcium looping offers a more effective route than remineralization for refurbishing Ni–CaO back to Ni–CaCO_3_, enabling improved CO_2_ conversion and syngas production in the subsequent DRM step.

### Stability Test of CaL-DRM

To evaluate the operational stability of the Ni–Ca material obtained via CO_2_ capture-mineralization, 10 cycles of isothermal test were conducted at 650 °C. Except for the first cycle, each cycle comprised a 15 min carbonation step followed by a 15 min DRM step, with a 2 min N_2_ purge in between. Figure 6 presents the stability performance results in terms of CO_2_ capture capacity (*X*_CO2_) during the carbonation step (i.e., excluding the amount of CO_2_ consumed via the reverse Boudouard reaction), and H_2_ (*Y*_H2_) and CO yields (*Y*_CO_) during the DRM step. In the first cycle, the fresh sample derived from CO_2_ capture-mineralization exhibited *Y*_H2_ and *Y*_CO_ values of ∼14.2 and ∼12.9 mmol/g_sample_, respectively. The slightly higher *Y*_H2_ value can be attributed to the occurrence of methane cracking. Note that the *Y*_H2_ value was lower in the first cycle than the subsequent cycles, likely due to the presence of Ni in its precursor form, where part of the generated H_2_ was consumed for in situ reduction to metallic Ni. In the second cycle, *X*_CO2_ value was ∼6.3 mmol/g_sample_ and maintained relatively stable throughout the cycles, reaching ∼6.1 mmol/g_sample_ in the 10th cycle. Meanwhile, both *Y*_H2_ and *Y*_CO_ values exhibited slight declines, reaching ∼14.5 and ∼12.1 mmol/g_sample_ in the 10th cycle, respectively. Throughout all the 10 cycles, no CO_2_ was detected in the product gas, indicating that CO_2_ was completely converted without being released from the Ni–Ca material.

**Figure 6:**
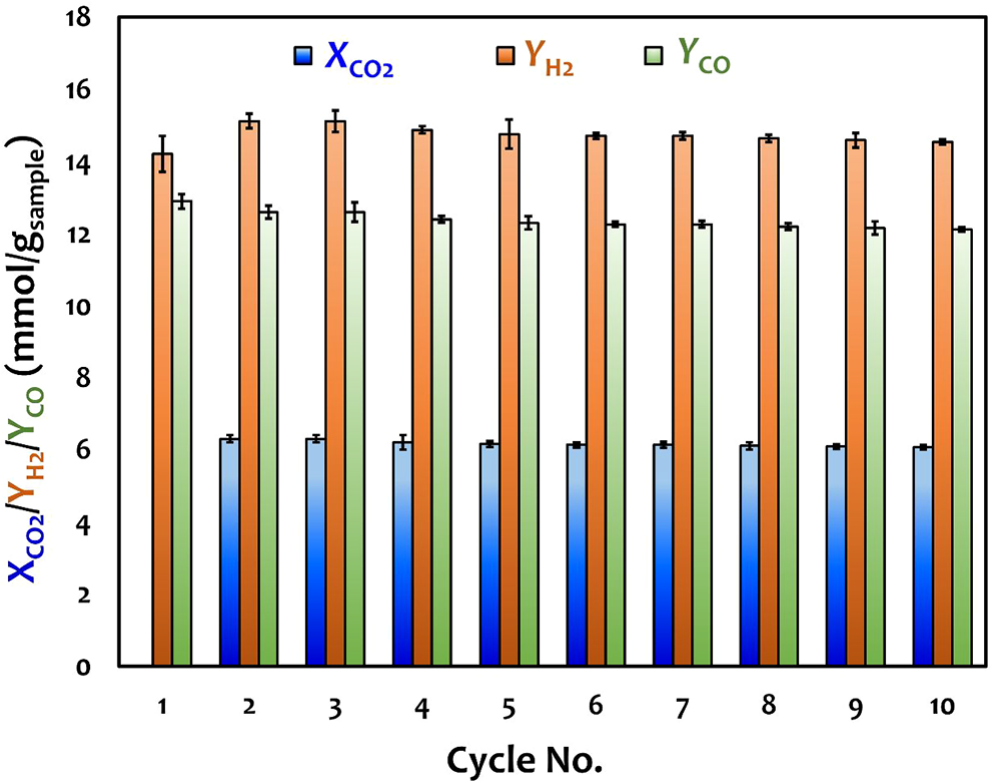
Cyclic performance efficiencies of the Ni–Ca material.

The mass balance of the stability test is summarized in Table S1 of the Supporting Information. Overall, the CO_2_ captured during the carbonation step was completely converted in the subsequent DRM step through its reaction with CH_4_. In the second step, methane participated in both the DRM and thermal decomposition, resulting in the formation of H_2_, CO and deposited carbon. One of the major challenges in a conventional DRM process is carbon deposition, which gradually lead to catalyst deactivation. However, by integrating the calcium looping process with DRM, the deposited carbon can be effectively removed in the subsequent carbonation step through the reverse Boudouard reaction with CO_2_. As evidenced in Table S1, CO was generated during each carbonation step, indicating the occurrence of the reverse Boudouard reaction, where CO_2_ reacted with the carbon deposited in the previous DRM step.

Material characterizations of the Ni–Ca material after the stability test are presented in Section S5 of the Supporting Information. XRD analysis (Figure S10) revealed the presence of CaO and Ca­(OH)_2_ phases, with no detectable CaCO_3_, confirming the complete decomposition of carbonates. Sintering of Ni crystallites was observed, with a *d*_c,Ni_ of 30.2 nm, attributed to prolonged exposure to high temperature during repeated cycles. Additionally, a diffraction peak at 2θ = 26° indicated the presence of crystalline carbon, resulting from coke formation via methane cracking. The Ni_3_C phase was not observable, attributable to the sufficiently high reaction temperature.^44,45^ SEM images (Figure S11) showed that the sample exhibited irregular morphology with an extent of agglomeration, attributable to the repeated phase transitions between CaCO_3_ and CaO. HR-TEM analysis (Figure 4c), along with the corresponding EDS mapping (Figure S3c), showed a well-maintained Ni–Ca interface and uniform Ni dispersion on the CaO matrix, confirming the high operational stability of the material. Nickel dispersion (*D*_metal_) and active metal surface area (*S*_msa_) of the sample were analyzed after the first and tenth DRM cycles using CO chemisorption. After the first DRM cycle, *D*_metal_ value was around 1.37% with a corresponding *S*_msa_ of 1.02 m^2^/g_sample_. After 10 cycles, these values decreased to approximately 0.78% and 0.58 m^2^/g_sample_, respectively, primarily due to the continuous phase transformations of the material throughout the cycles.

To evaluate the performance of our mineralized Ni–Ca material, a comparison was made with the previously reported values of CaO–Ni-based bifunctional materials for CaL-DRM, as summarized in Table 1. Overall, our material demonstrated sufficiently high CO_2_ conversion (>99%) and a stable H_2_/CO ratio of ∼1.1 at a relatively moderate temperature of 650 °C. These results highlight the effectiveness of using mineralized CaCO_3_ as a sorbent, incorporated with Ni, for integrated CO_2_ capture and conversion.

**Table 1:** Comparison of the Catalytic Performances of CaO–Ni Based Bifunctional Materials for CaL-DRM Reported in the Literatures

sample	temperature (°C)	CO_2_ conversion during DRM (%)	H_2_/CO ratio during DRM	refs
Ni–Ca	650	>99	1.1	this study
CaO-0.3Ni/0.2CeO_2_	600	97.2	1.2	^24,35^
CaO-0.05Ni-0.05CeO_2_	680	47	1.7	^23^
NiCa_85_Ce_15_	650	>90	∼0.9	^25^
NiCe/Ca@Zr	720	85	N/A	^30^
CaO/Ni_9	800	65	∼1	^31^

## Conclusions

This study demonstrates the feasibility of integrating CO_2_ capture-mineralization with the calcium looping-methane dry reforming process for efficient CO_2_ capture and utilization. The experimental results confirmed that CO_2_ captured using MEA can be effectively recovered through mineralization, forming CaCO_3_, which can then be incorporated with Ni to serve as a dual-functional material. During CaL-DRM, the DRM step facilitated syngas production while enabling in situ CaO regeneration. In the subsequent carbonation step, the material successfully reabsorbed CO_2_, allowing for its cyclic utilization. A 10-cycle stability test confirmed that the Ni–Ca material maintained stable CO_2_ uptake and syngas yields with minimal performance loss, and no CO_2_ was released throughout the cycles. Overall, this study provides a promising pathway for sustainable CO_2_ capture and utilization, offering an energy-efficient alternative to conventional industrial processes.

## Supplementary Material



## Notes

Dimensionless number; experimental setup; additional SEM and TEM images; CO_2_-TPD and CH_4_-based temperature-programmed reaction profiles; additional information of remineralization route; additional information of stability test (PDF)
