# Supplementary material for: CO2 Capture-Mineralization for Calcium-Looping Integrated with Methane Dry Reforming
Source: Langmuir. 2025 Sep 8;41(36):24398–407. doi: 10.1021/acs.langmuir.5c02551 (PMC12444975; doi:10.1021/acs.langmuir.5c02551)
Supplement: Supplementary file 1 [file la5c02551_si_001.pdf]

Supporting Information

CO_2 Capture-Mineralization for Calcium-Looping Integrated with Methane Dry Reforming

Zhi Xuan Law[1], *Nattanan Watcharasawat*[2], *Varong Pavarajarn*[2,*], *De-Hao Tsai*[1,*]

[1] Department of Chemical Engineering, National Tsing Hua University, Hsinchu, Taiwan, R.O.C.

[2] Department of Chemical Engineering, Chulalongkorn University, Bangkok, Thailand

[*] To whom correspondence should be addressed. E-mail: dhtsai@mx.nthu.edu.tw; fchvpv@eng.chula.ac.th

Table of Content

1. Estimations of dimensionless numbers during CO_2 absorption process
2. Description of the experimental setup
3. Additional material characterizations of the Ni-Ca material
4. Additional information of the re-mineralization route
5. Additional information of stability test

1. Estimations of dimensionless numbers during CO_2 absorption process

In our study, the mass transfer performance during CO_2 absorption into MEA was primarily governed by liquid-phase transport and rapid chemical reaction. Given the stirred-tank setup at room temperature, we approximated the **Reynolds number (*Re*)** using Eq. (S1), obtaining a value of around **600**, which indicates a laminar flow.[1]

$$Re = \frac{\rho \cdot N \cdot D^2}{\mu} \qquad (S1)$$

ρ (fluid density of 5 M MEA) = ~1020 kg/m^3

N (stirring speed) = 2.5 rps

D (stirrer diameter) = 0.03 m

μ (dynamic viscosity of 5 M MEA) = ~0.0038 kg/m·s

Given that the **Schmidt number (*Sc*)** for CO_2 in water at room temperature is known to be approximately 410,[2] the *Sc* value for CO_2 in 5 M MEA is estimated to be around **1520** using Eq. (S2).[1] This high *Sc* number suggests that mass transfer is the rate-limiting step in the CO_2 absorption process, which aligns with the typical behavior in gas-liquid absorption systems where CO_2 must diffuse through the liquid film before chemical absorption occurs. Hence, enhancing mass transfer efficiency (i.e., through effective mixing) is critical for improving CO_2 absorption.

$$Sc_{MEA} = \frac{\mu}{\rho \cdot D_{AB}} = Sc_{water} \cdot \frac{(\frac{\mu}{\rho})_{MEA}}{(\frac{\mu}{\rho})_{water}} \qquad (S2)$$

ρ_{water} = 997 kg/m^3

μ_{water} = 0.001 kg/m·s

Sherwood number (Sh) can be related to *Re* and *Sc* via empirical correlations ($Sh \approx 2 + 0.6\, Re^{1/2} Sc^{1/3}$),[1] which provides a *Sh* value of approximately **170**, indicating that convective mass transfer (i.e., from stirring and bulk flow) dominates over pure diffusion. This again emphasizes the importance of sufficient mixing for enhancing CO_2 mass transfer in the MEA solution.

2. Description of the experimental setup

Figure S1a shows the experimental setup, which was composed of a fixed-bed reactor system (right) and a downstream detector system (left). A U-shape quartz tube (22 cm in length and 1 cm in inner diameter), acting as the fixed-bed reactor, was inserted into a temperature-programmed tube furnace (P961; Shiuan Rong Industrial Co., Ltd, Taipei, Taiwan, ROC). As shown in Figure S1b, the temperature was ramped at 10 °C/min from room temperature to 600 °C for the carbonation step, and to 800 °C for the DRM step. The flow rates of the carrier gas and the reactant gases were controlled by the mass flow controllers (5800E Series; Brooks Instrument, PA, USA). During DRM, 5 vol% CH_4/N_2 was introduced. After completion, the system was purged with N_2 for 2 minutes before introducing 5 vol% CO_2/N_2 for the carbonation step. The outlet gaseous concentrations were measured using the following instruments connected in series: a CO_2 non-destructive infrared spectrometer (ND-IR; CI-IR 20; Chang-Ai, Shanghai, China), a CH_4 ND-IR (CI-IR 10; Chang-Ai), a CO ND-IR (CI-IR 10; Chang-Ai) and a H_2 thermal conductivity detector (TCD; CI-TC 90; Chang-Ai).

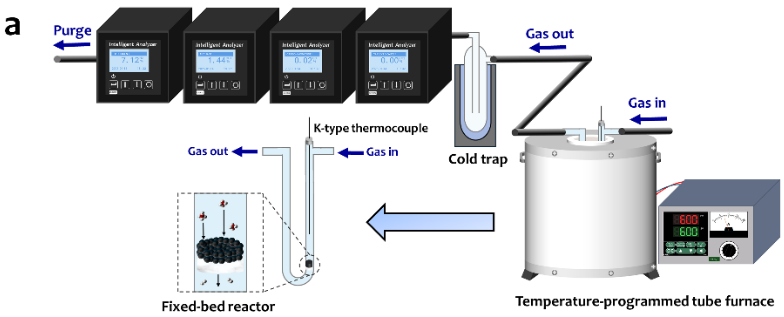

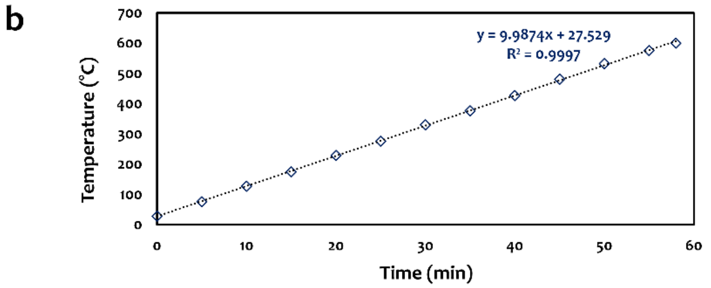

Figure S1. (a) Schematic diagram of a temperature-programmed reaction platform, which was composed of a fixed-bed reactor system (right) and a downstream detector system (left) having a series of ND-IR spectrometers (CH_4, CO_2, CO) and a H_2-based TCD. (b) Temperature versus time graph.

3. Additional material characterizations of the Ni-Ca material

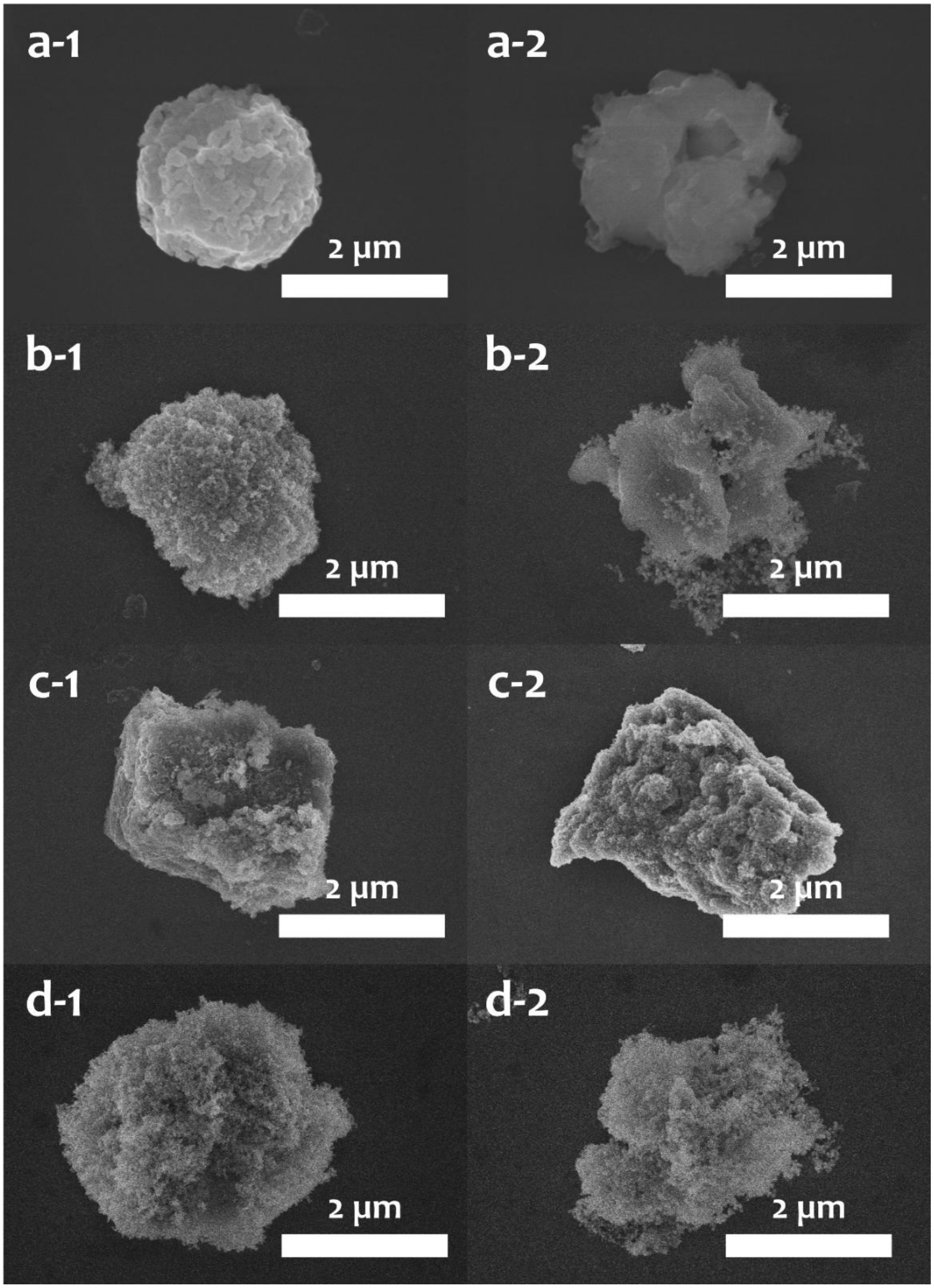

Figure S2. SEM images of the Ni-Ca material. (a) $CaCO_3$ after mineralization. (b) Ni-Ca after first DRM step. (c) Ni-Ca after carbonation step. (d) Ni-Ca after second DRM step. The scale bars are 2 µm.

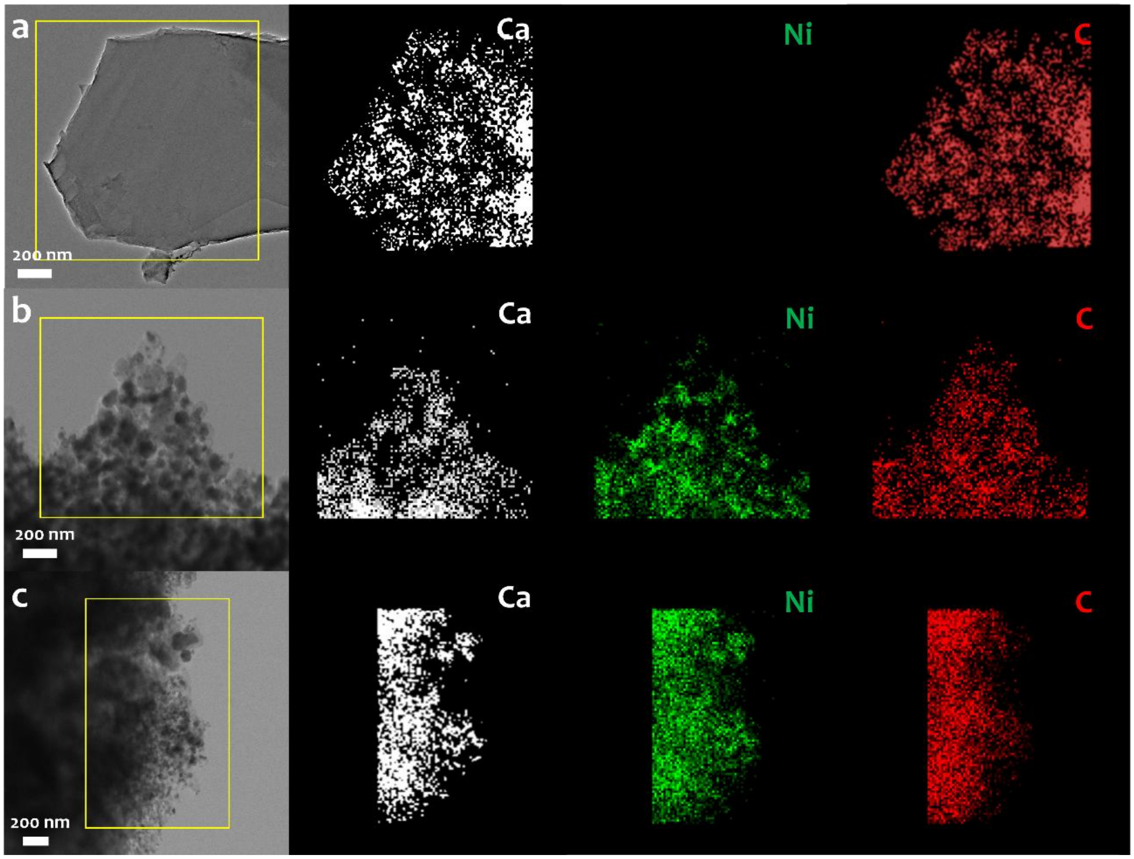

Figure S3. TEM-EDS images of the representative Ni-Ca material before and after activity test. (a) $CaCO_3$ after mineralization. (b) Ni-Ca material after first DRM step. (c) Ni-Ca material after 10-cycle stability test. The scale bars are 200 nm.

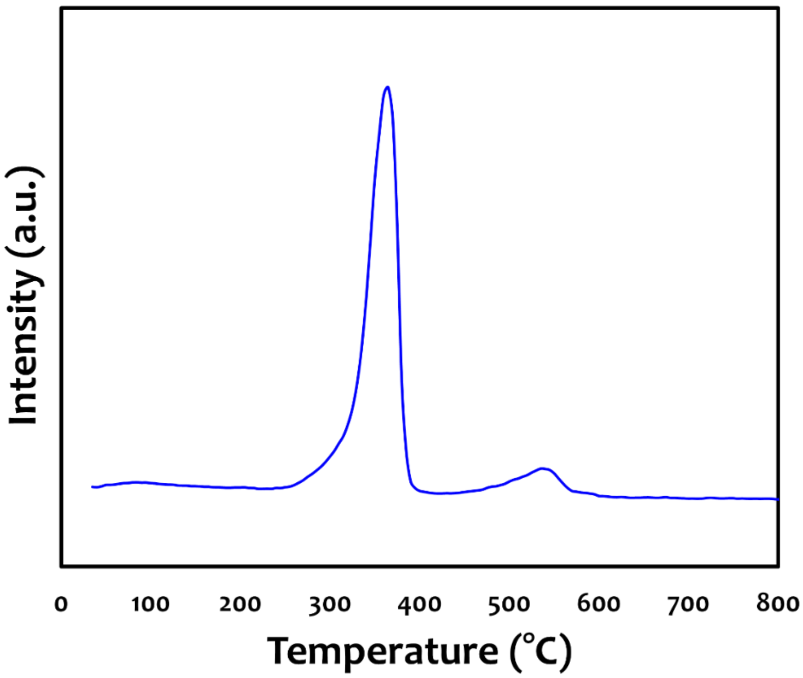

Figure S4. CO_2-TPD profile of Ni-Ca material after first DRM step.

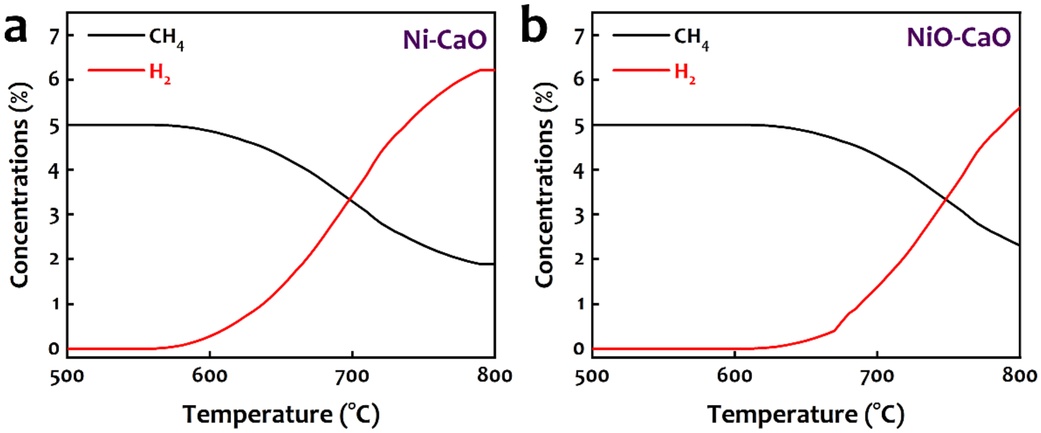

Figure S5. The profiles of CH_4-based temperature-programmed reaction of (a) Ni-CaO and (b) NiO-CaO.

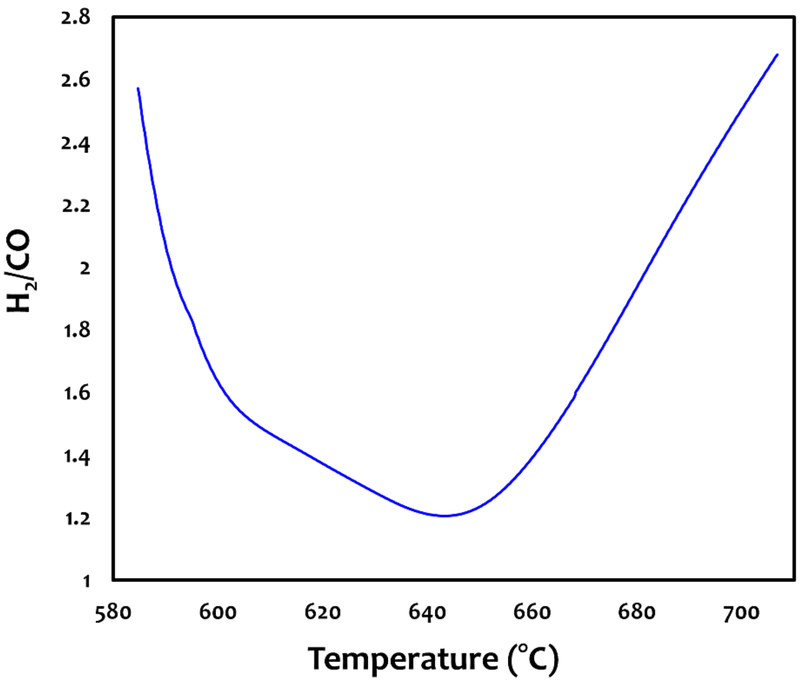

Figure S6. H_2/CO ratio during DRM.

4. Additional information of the re-mineralization route

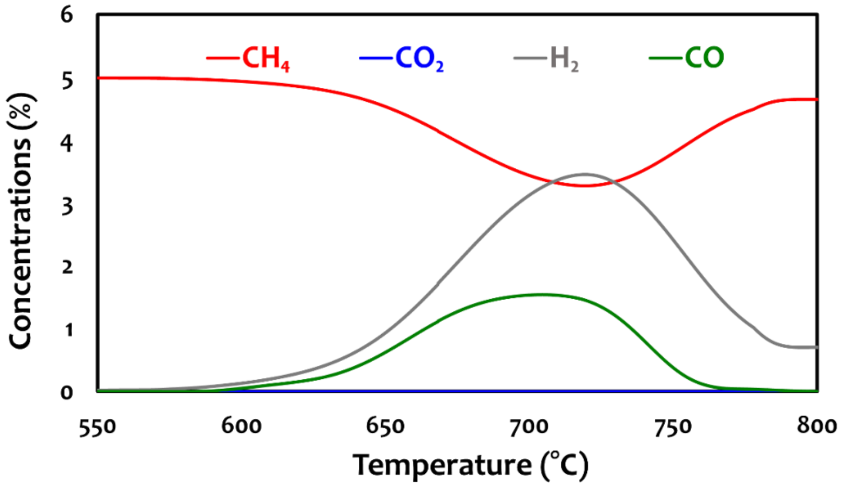

Figure S7. Gaseous concentration profiles during temperature-programmed DRM using re-mineralized Ni-Ca material.

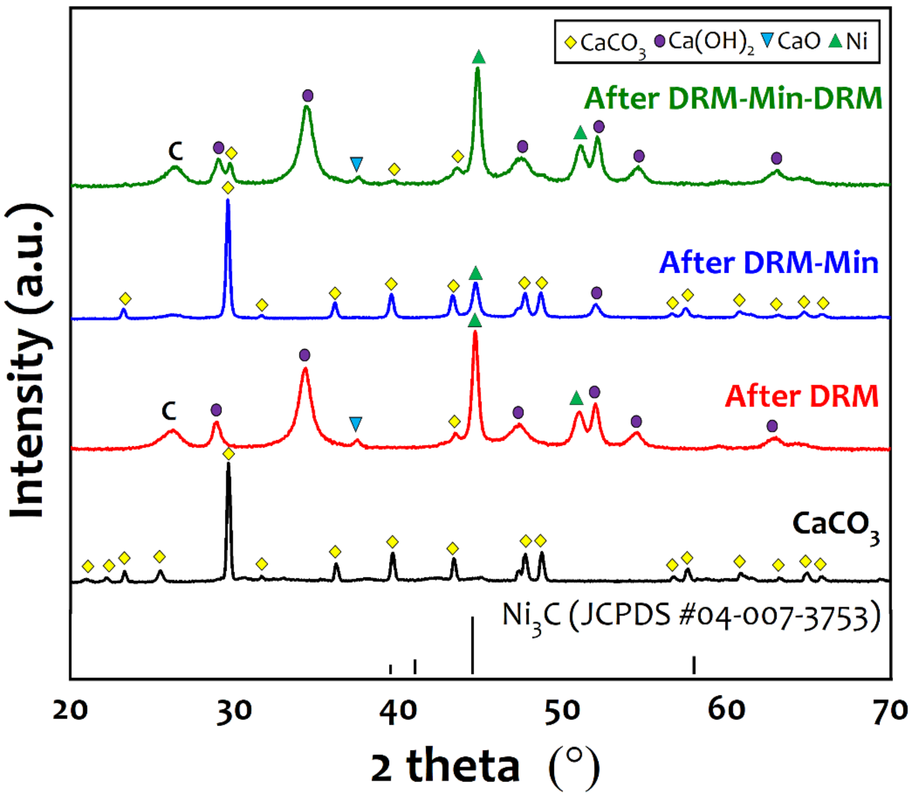

Figure S8. XRD pattern of Ni-Ca material before and after temperature-programmed activity test via the mineralization route.

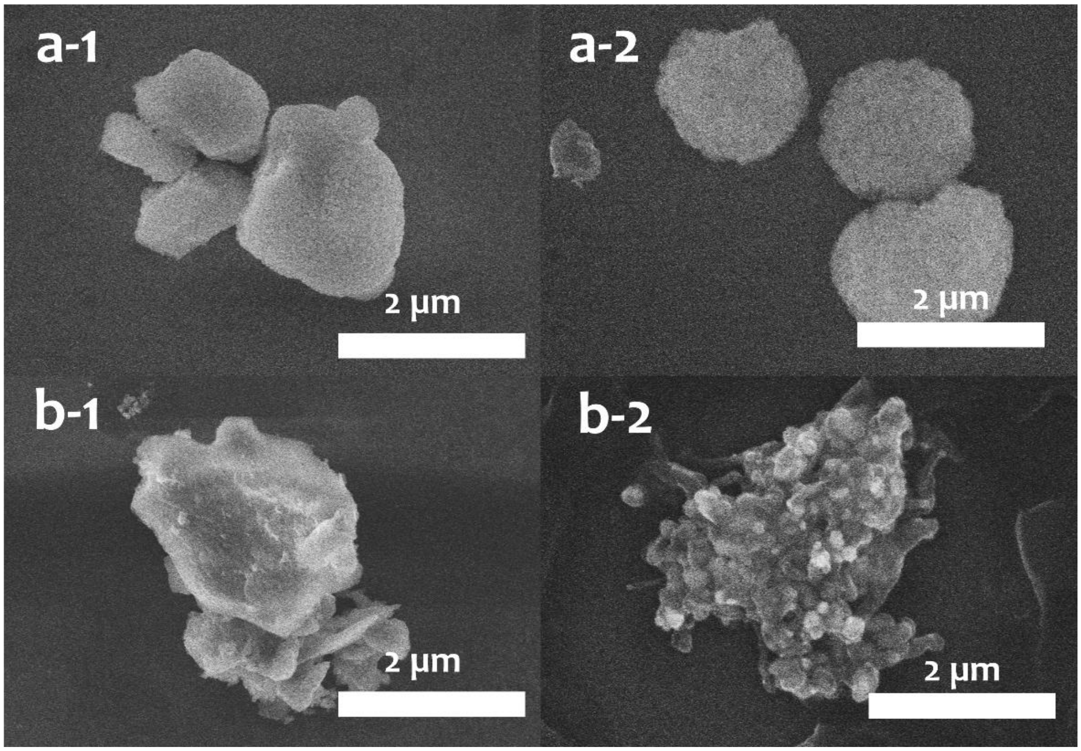

Figure S9. SEM images of the Ni-Ca material. (a) Ni-Ca after re-mineralization. (b) Ni-Ca after re-mineralization followed by DRM. The scale bars are 2 μm.

5. Additional information of stability test

Table S1. Mass balance of the cyclic CaL-DRM test.

Cycle	1st step: Carbonation			2nd step: DRM			
	**CO_2 Uptake (mmol/g)**	**CO_2 Converted[a] (mmol/g)**	**CO Released[a] (mmol/g)**	**CH_4 Converted (mmol/g)**	**H_2 Generated (mmol/g)**	**CO Generated (mmol/g)**	**Deposited carbon[b] (mmol/g)**
**1**	-	-	-	7.8 ± 0.20	14.2 ± 0.49	12.9 ± 0.20	
**2**	6.3 ± 0.10	1.2 ± 0.07	2.4 ± 0.09	7.6 ± 0.41	15.1 ± 0.21	12.6 ± 0.18	1.3
**3**	6.3 ± 0.12	1.3 ± 0.08	2.4 ± 0.12	7.6 ± 0.31	15.1 ± 0.30	12.6 ± 0.27	1.3
**4**	6.2 ± 0.20	1.2 ± 0.04	2.2 ± 0.06	7.4 ± 0.22	14.9 ± 0.11	12.4 ± 0.09	1.2
**5**	6.2 ± 0.08	1.3 ± 0.10	2.3 ± 0.21	7.4 ± 0.18	14.8 ± 0.40	12.3 ± 0.18	1.3
**6**	6.1 ± 0.07	1.4 ± 0.07	2.4 ± 0.12	7.4 ± 0.07	14.7 ± 0.08	12.3 ± 0.07	1.2
**7**	6.1 ± 0.08	1.1 ± 0.06	2.3 ± 0.14	7.4 ± 0.04	14.7 ± 0.10	12.3 ± 0.09	1.2
**8**	6.1 ± 0.10	1.3 ± 0.08	2.4 ± 0.16	7.3 ± 0.09	14.6 ± 0.13	12.2 ± 0.09	1.2
**9**	6.1 ± 0.05	1.5 ± 0.07	2.8 ± 0.20	7.3 ± 0.11	14.6 ± 0.20	12.2 ± 0.18	1.2
**10**	6.1 ± 0.06	1.4 ± 0.12	2.6 ± 0.21	7.3 ± 0.05	14.5 ± 0.07	12.1 ± 0.06	1.3

[a]*reverse Boudouard reaction ($CO_2 + C \rightarrow 2CO$)*

[b]*by calculation using carbon balance*

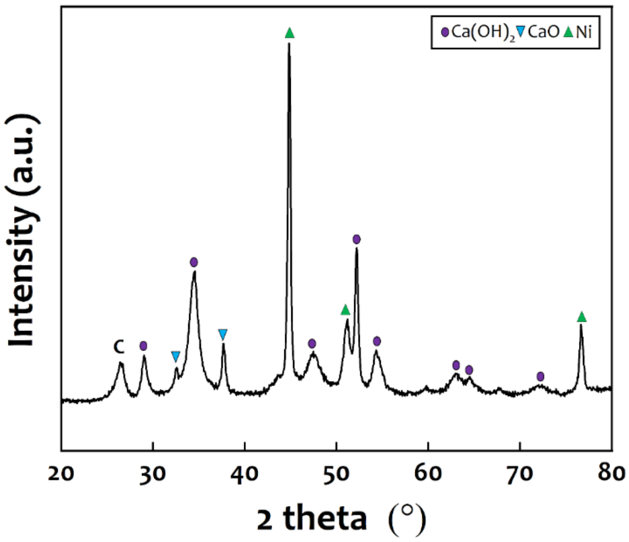

Figure S10. XRD pattern of the Ni-Ca material after 10-cycle stability test at 650 °C.

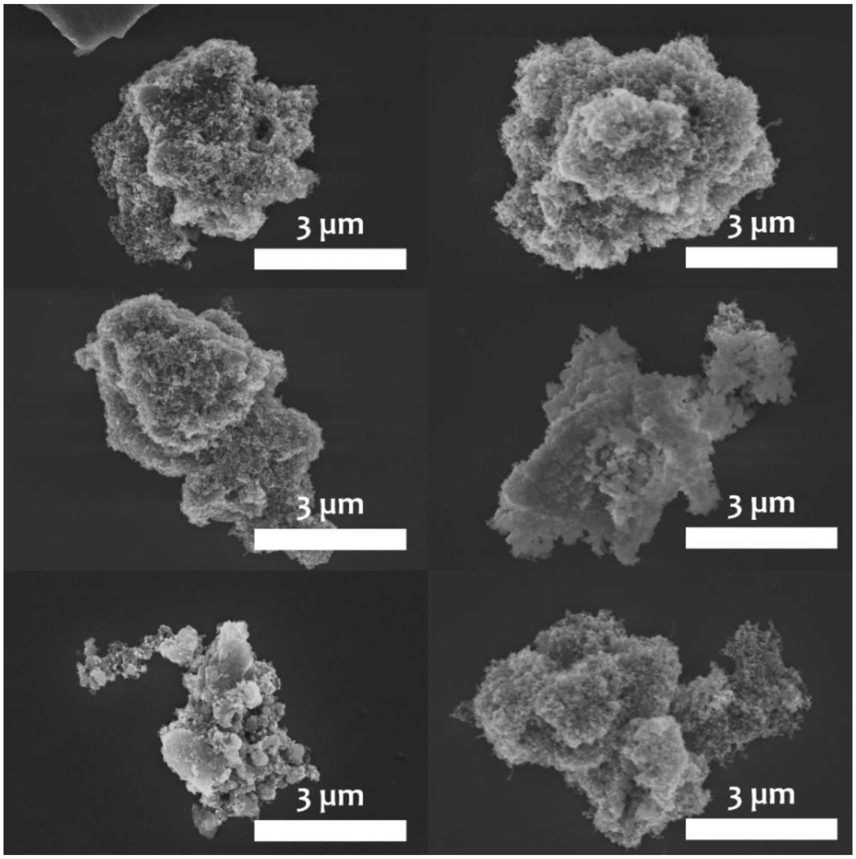

Figure S11. SEM images of the Ni-Ca material after 10-cycle stability test at 650 °C.

The scale bars are 3 μm.

References

(1) Bird, R. B.; Stewart, W. E.; Lightfoot, E. N. *Transport Phenomena*; Wiley, 2006.

(2) Gualtieri, C.; Angeloudis, A.; Bombardelli, F.; Jha, S.; Stoesser, T. On the values for the turbulent Schmidt number in environmental flows. *Fluids* **2017**, *2* (2), 17.
